# The parietal cortex and saccade planning: lessons from human lesion studies

**DOI:** 10.3389/fnhum.2013.00254

**Published:** 2013-06-07

**Authors:** Radek Ptak, René M. Müri

**Affiliations:** ^1^Division of Neurorehabilitation, University Hospitals GenevaGeneva, Switzerland; ^2^Laboratory of Cognitive Neurorehabilitation, Faculty of Medicine, University of GenevaGeneva, Switzerland; ^3^Faculty of Psychology and Educational Sciences, University of GenevaGeneva, Switzerland; ^4^Division of Cognitive and Restorative Neurology, Department of Neurology, University HospitalInselspital, Bern, Switzerland

**Keywords:** parietal lobe, intraparietal sulcus, saccade planning, visual attention, eye movements, spatial neglect, simultanagnosia, Bálint's syndrome

## Abstract

The parietal cortex is a critical interface for attention and integration of multiple sensory signals that can be used for the implementation of motor plans. Many neurons in this region exhibit strong attention-, reach-, grasp- or saccade-related activity. Here, we review human lesion studies supporting the critical role of the parietal cortex in saccade planning. Studies of patients with unilateral parietal damage and spatial neglect reveal characteristic spatially lateralized deficits of saccade programming when multiple stimuli compete for attention. However, these patients also show bilateral impairments of saccade initiation and control that are difficult to explain in the context of their lateralized deficits of visual attention. These findings are reminiscent of the deficits of oculomotor control observed in patients with Bálint's syndrome consecutive to bilateral parietal damage. We propose that some oculomotor deficits following parietal damage are compatible with a decisive role of the parietal cortex in saccade planning under conditions of sensory competition, while other deficits reflect disinhibition of low-level structures of the oculomotor network in the absence of top-down parietal modulation.

## Introduction

The parietal cortex is a region of convergence for multiple sensory inputs from the visual, auditory and somatosensory modality, and recurrent pathways to and from the premotor and lateral prefrontal cortex. A central part of its activity is dedicated to the orienting and maintenance of spatial attention and the generation and control of saccadic eye movements. Several parietal areas lying within the intraparietal sulcus (IPS) of the monkey brain show activity related to the planning and execution of saccades (Colby and Goldberg, 1999). Some of the neurons located in these areas signal the onset of a visual stimulus that has previously been defined as saccade target (Bushnell et al., 1981; Gottlieb et al., 2005). Others show visuomotor activity, which can be observed when a visual target appears, but the monkey is instructed to maintain fixation until a go-signal is presented (Colby and Duhamel, 1991). In this situation visuomotor neurons produce a rapid burst of spikes following the onset of the target, followed by a fast decrease and again a gradual increase of activity culminating with another burst prior to saccade initiation. In addition to visual and visuomotor activity, some parietal neurons are particularly responsive when the monkey is actively fixating a stimulus. Interestingly, most of these cells show enhanced activity for relevant as compared to irrelevant stimuli, but irrespective of the specific features of the stimulus (Bushnell et al., 1981; Constantinidis and Steinmetz, 2001) suggesting that these cells discriminate targets from distracters based on a feature-independent representation (Gottlieb, 2012).

**Figure 1:**
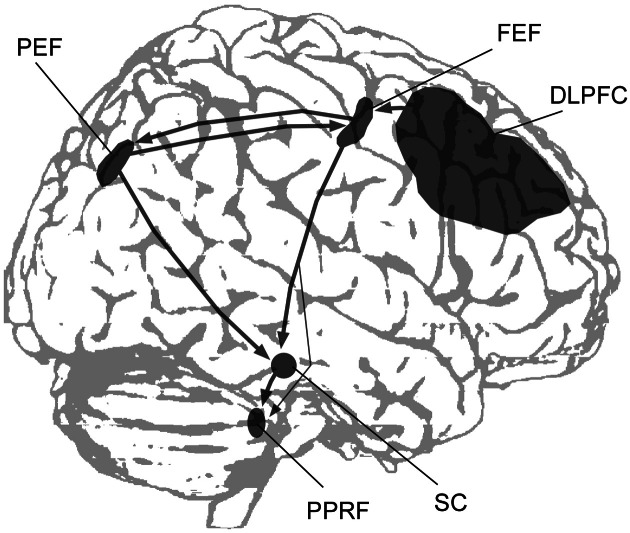
**A simplified scheme showing the main cortical regions and subcortical structures involved in the control of saccadic eye movements (DLPFC, dorsolateral prefrontal cortex; FEF, frontal eye field; PEF, parietal eye field; PPRF, paramedian pontine reticular formation; SC, superior colliculus)**.

Visual, visuomotor and fixation activity is predominant in area 7a (whose human homologue is probably the angular gyrus) and the lateral intraparietal area (LIP), whose homologue in humans has been termed the parietal eye field (PEF; Figure 1). Functional imaging studies have localized the PEF in the posterior IPS (Müri et al., 1996; Culham and Kanwisher, 2001; Pierrot-Deseilligny et al., 2004). This region is highly active when subjects execute saccadic eye movements, or when they shift their attention without shifting the gaze, making it difficult to distinguish between mechanisms involved in saccade planning and the orienting of attention (Corbetta et al., 1998; Perry and Zeki, 2000). In saccade tasks the PEF is activated together with the frontal eye field (FEF; see Grosbras et al., 2005 for a review of functional imaging studies), which is located in dorsal premotor cortex (Paus, 1996). Fronto-parietal connections between the posterior parietal and premotor/prefrontal cortex form a network that is involved in the filtering of sensory contents and the covert and overt guidance of spatial attention (Gottlieb, 2007; Corbetta et al., 2008; Ptak, 2012). The PEF and FEF both have direct and independent connections to the superior colliculus, which is the primary mesencephalic structure playing a crucial role in saccade initiation and the maintenance of fixation (Wurtz and Mohler, 1976; Munoz and Wurtz, 1992). The cortical saccade network is thus directly linked to mesencephalic centers that trigger the execution of saccades (Figure 1).

The present paper discusses the effects of focal unilateral or bilateral parietal damage on saccade planning as determined by experimental studies using relatively simple saccade paradigms and investigations of eye movements during the exploration of complex visual scenes. The focus of this review is (a) to describe the effect of parietal damage on basic parameters affecting saccade timing and metrics, (b) to compare the impact of unilateral damage (leading to spatial neglect when the lesion affects the right hemisphere) with the effects of bilateral damage to the posterior parietal lobe (resulting in Bálint's syndrome), and (c) to relate the deficits of saccade programming following parietal damage to underlying dysfunctions of high-level cognitive functions or low-level oculomotor processes.

## Experimental paradigms for eye movement research

Saccades are rapid eye movements that have a latency of 200–250 ms following the onset of a peripheral target (Ramat et al., 2007). Mechanisms of saccade planning are often investigated using basic paradigms in which participants execute saccades toward the visual periphery under different experimental conditions that favor voluntary, reflexive or memory-dependent processing (Findlay and Gilchrist, 2003; Müri and Nyffeler, 2008). Based on these studies, several models of saccade planning distinguish between processes involved in the computation of saccade amplitude (“where”-processes) and processes involved in the timing of saccades (“when”-processes; Findlay and Walker, 1999; Girard and Berthoz, 2005; Ramat et al., 2007). This distinction is justified by the observation that some experimental variables affect the timing of saccades without affecting their amplitude and vice versa. Thus, the offset of the central fixation stimulus 150–200 ms prior to the appearance of the peripheral target shortens saccade latencies by 20–30 ms as compared to when the fixation stimulus overlaps with the upcoming target. This phenomenon is known as the *gap effect* or *fixation-offset effect* (Saslow, 1967). The gap-effect has been related to inhibitory interactions between collicular fixation neurons and saccadic burst neurons, which show opposite activity during fixation and prior to a saccadic eye movement (Munoz and Wurtz, 1992, 1993; Dorris and Munoz, 1995; Gandhi and Keller, 1999).

A second factor that affects saccade latencies has been known as the *remote distracter effect*: a distracter stimulus presented in the visual field opposite the target increases saccade latencies (Walker et al., 1995, 1997). While the gap effect and the remote distracter effect influence temporal parameters without affecting saccade metrics, the opposite is seen in the *global effect*: a redundant stimulus presented in the same hemifield as the target leads to normal-latency saccades, but a modification of saccade amplitude because the saccade lands between the two stimuli (Findlay, 1982). A more complicated saccade paradigm is the *antisaccade task*, in which subjects are asked to make a saccade to the location opposite the upcoming target (Hallett, 1978). The antisaccade task measures the ability of participants to inhibit a reflexive saccade toward the visual target in favor of a voluntary saccade in the opposite direction (Munoz and Everling, 2004). Thus, this task opposes voluntary and reflexive processes involved in saccade planning, which makes it particularly interesting for the study of the neural correlates and cognitive mechanisms that modulate oculomotor responses.

Some of these experimental effects operate at relatively low levels in the oculomotor circuitry. This is particularly the case for the gap effect, which has been related to the activity of opponent neural mechanisms involved in fixation and the release of saccades in the superior colliculus (Dorris and Munoz, 1995). Similarly, the remote distracter effect can be explained by low-level inhibitory interactions between two competing neuronal populations activated by the target and the distracter in a collicular saliency map (Walker et al., 1997). Thus, it is possible to study basic parameters affecting saccade programming in the laboratory with specific experimental paradigms. However, these paradigms all use relatively simple visual displays composed of one or two stimuli on a homogenous background, which are generally not encountered in everyday life. Real-life situations frequently require the exploration of complex visual scenes in which the target is not known in advance and where subjects often produce long sequences of saccades (Henderson and Hollingworth, 1999; Tatler et al., 2011). Eye movements involved in the scanning of naturalistic scenes have been investigated in several recent studies involving patients with parietal damage, and the findings of these studies give important clues about the role of this brain region in saccade target selection in complex visual displays.

In this review we first examine findings of studies that tested patients with unilateral focal brain damage using basic saccade paradigms before discussing results obtained with more complex visual exploration tasks. We then evaluate findings from studies on patients with bilateral parietal damage.

## Impairments of saccade programming following unilateral parietal damage

### Eye movements in basic saccade paradigms

In one of the first systematic studies examining the programming of saccades to targets shown in the left or right visual hemifield, Pierrot-Deseilligny et al. (1991) tested ten patients with circumscribed lesions to the PPC using a gap paradigm. Compared to healthy controls and patients with damage to prefrontal cortex or the FEF the parietal patients had markedly increased saccade latencies. Interestingly, while patients with left PPC damage only had increased latencies for contralateral (right) saccades, right PPC damage resulted in a bilateral latency increase. Braun et al. (1992) also found that patients with dorsal parietal damage had increased saccade latency. In addition, they also reported high latency variability and a significant reduction of the numbers of very fast saccades (so-called express saccades). Pierrot-Deseilligny et al. (1991) concluded from their findings that the PPC (and the PEF in particular) is crucially involved in the programming of reflexive saccades, which are generated upon appearance of sudden, unexpected visual stimuli. This conclusion is in agreement with more recent functional imaging and event-related potential studies of voluntary and reflexive saccades (Grosbras et al., 2005; Ptak et al., 2011). Furthermore, a study examining the effects of parieto-collicular disconnection on voluntary and reflexive saccades supports the importance of the parietal cortex in saccade programming to unpredictable targets. Gaymard et al. (2003) localized the direct pathway connecting the LIP with the superior colliculus in the monkey and found that it travelled through the posterior portion of the posterior limb of the internal capsule. They reported that the accuracy of saccades in patients with small infarcts to this region was affected only in spatially unpredictable conditions, in which saccadic responses are guided by external signals. In contrast, performance was normal when the target location was predictable and saccades were triggered voluntarily.

Unfortunately, these early anatomical studies do not inform us about the cognitive deficits that might be crucially associated with impaired saccade programming. The neuropsychological syndrome that has historically been associated with parietal damage is spatial neglect, a disorder characterized by unawareness of visual, auditory or tactile stimuli presented in space or to the body side contralateral to the brain damage (Vallar and Perani, 1986; Mort et al., 2003; Husain and Nachev, 2006; Golay et al., 2008). However, the majority of neglect patients have large lesions that cover substantial parts of the parietal, frontal and temporal lobes, and some studies found the region of greatest overlap outside the parietal cortex (Karnath et al., 2004) or in subcortical white matter (Doricchi and Tomaiuolo, 2003). The current interpretation is therefore that neglect results from damage or dysfunction of a fronto-parieto-temporal network, and that some neglect symptoms are associated with specific lesion foci within this network (Corbetta and Shulman, 2011; Chechlacz et al., 2012; Karnath and Rorden, 2012; Ptak, 2012). We have recently found that among several areas belonging to the cortical attention network, only the posterior intraparietal sulcus (the region where the PEF is located) was a critical predictor of distinct contralateral attention deficits in neglect (Ptak and Schnider, 2011), supporting a central role of the posterior parietal cortex in dynamic aspects of spatial attention (i.e., computation of attentional priority, attention shifting and disengagement; Vandenberghe et al., 2012). Unfortunately, the association between eye movement disorders and brain damage in patients with neglect has not been systematically evaluated in previous lesion studies (a notable exception being a study by Mannan et al., 2005 who found no association of ocular re-fixation behavior with specific cortical damage in neglect). Thus, though the link between neglect and parietal damage is a matter of debate, given the close anatomical and functional relationship between spatial attention and saccade programming, much of our review focuses on impairments of saccade planning in spatial neglect.

Neglect patients typically show a spontaneous ipsilateral deviation of the eyes which becomes even more evident when they actively explore their environment or search for an object (Karnath and Rorden, 2012). An early study of saccade performance in neglect (Girotti et al., 1983) showed that patients were unaware of contralesional targets in one quarter of all trials and consequently did not perform any eye movement in these trials. When they detected a contralesional target patients produced a staircase pattern of multiple hypometric long-latency saccades before their gaze reached the target. The increase of contralateral saccade latency was confirmed by later studies, though they did not specifically investigate the gap effect or remote distracter effect (Karnath et al., 1991; Behrmann et al., 2001). A first systematic study of neglect patients' eye movements in the gap and overlap task was performed by Walker and Findlay (1996). Out of their four neglect patients only two made left saccades, and both showed saccade latencies that were significantly increased in the overlap condition. However, these patients showed relatively slight neglect in classical neglect tests, and it is unclear to what extent their lesions affected the parietal lobe. In order to study systematically the effect of parietal neglect on saccades we measured saccade amplitude and latency of seven patients with moderate to severe left neglect in a task requiring the execution of saccades to targets at two different eccentricities in the left or right hemifield (Ptak et al., 2007). The target was either presented alone or simultaneously with a distracter appearing at fixation, in the same hemifield as the target or in the opposite hemifield. Thus, the paradigm allowed for quantifying the remote distracter effect when the distracter was presented in the opposite hemifield, the global effect when it was shown in the same hemifield as the target, and the gap/overlap effects when it appeared at fixation.

**Figure 2:**
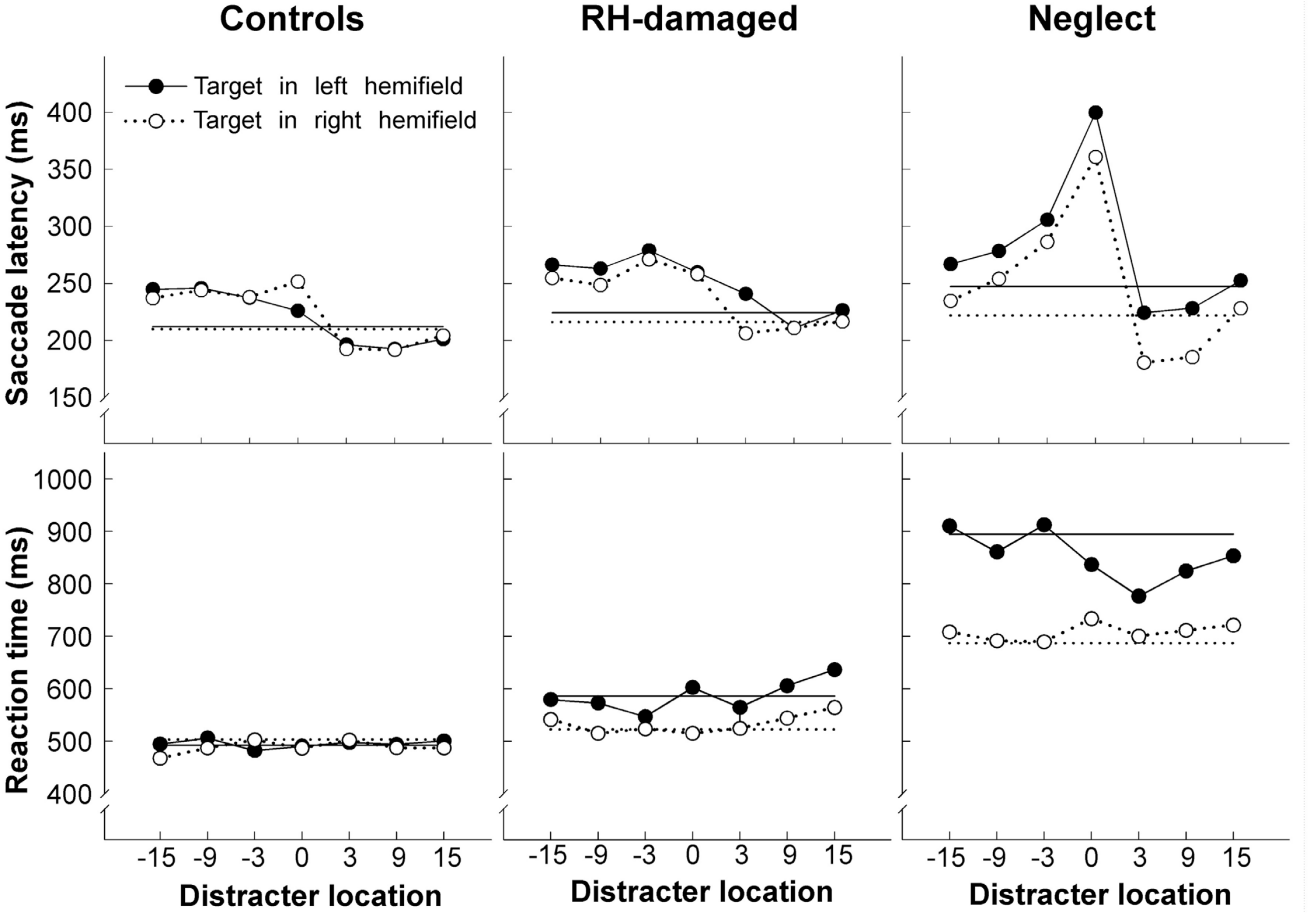
**Average latency of saccades (upper row) and manual reaction times (lower row) as a function of distracter location, shown separately for healthy controls, brain injured patients without neglect, and neglect patients**. Positive numbers indicate distracter locations (in degrees) in the same hemifield as the target (for left targets distracters in the left hemifield are shown on the right side and distracters in the right hemifield on the left side of each graph). The straight horizontal lines show average latency/reaction time when no distracter was presented. Note the increase of saccade latency for neglect patients when the distracter was presented at fixation (position 0). Figure adapted from Ptak et al. (2007) by permission from Oxford University Press.

Compared to healthy controls and control patients with right-hemisphere lesions neglect patients had only slightly increased saccade latencies for contralesional targets when no distracter was present (Figure 2). However, the pattern of response changed when a distracter was added: distracters in the right hemifield strongly captured the gaze of neglect patients, resulting in up to 60% directional errors. The capture of gaze by ipsilesional distracters is a robust finding and has been observed in patients with post-acute neglect (Müri et al., 2009) as well as in clinically recovered neglect (Harvey et al., 2002; Olk et al., 2002; Pflugshaupt et al., 2004). More interestingly, in those trials where no directional error was made remote distracters did not increase contralesional saccade latencies of neglect patients more than of control participants. These results suggest that an ipsilesional distracter interferes at two different levels with saccade programming: it either interacts with processes involved in the selection of the saccade target (the where-process; eventually resulting in a directional error) or it delays the onset of the saccade once the correct target has been selected (when-process). The first type of interference appears to be pathologically increased in spatial neglect, while the second type is unaffected.

A further finding of our study relates to the effect of the distracter presented at fixation. Control participants showed an increase of saccadic latency of approximately 20% when the distracter appeared at fixation (overlap) compared to when there was no distracter (gap). In contrast, the increase averaged more than 60% in neglect patients, and this pathological latency increase was comparable for saccades directed to the left (impaired) or the right (intact) hemifield. A foveal distracter had no influence on performance when patients were asked to respond manually (by pressing a response button) upon detection of the peripheral target (Figure 2). Thus, this study shows that some characteristics of saccades in parietal neglect following right focal brain damage are unilateral (capture of gaze by ipsilesional distracters), whereas other features are bilateral (exaggerated overlap-effect). The latter finding is compatible with other observations of bilateral effects of unilateral parietal damage on saccades. Butler et al. (2006) reported a single patient with a small cortical lesion located at the temporo-parietal junction, who showed exaggerated capture of gaze by irrelevant abrupt-onset distracters, whether these appeared in the left or right hemifield. At the time of the study the patient had only slight left hemineglect. In another study Butler et al. (2009) tested the performance of 13 neglect patients in the antisaccade task. The patients produced a similarly high proportion of errors for antisaccades directed to the left (75% errors) as for antisaccades directed to the right hemifield (65% errors). The authors proposed that this pattern might reflect two different factors: according to their interpretation neglect patients fail to suppress saccades to right-sided targets due to their spatial attention bias. In contrast, for left-sided targets and antisaccades to the right hemifield, they proposed that patients failed to compute the inversed vector necessary for the specification of the spatial coordinates for the antisaccade because of a deficient coding of target location. Their interpretation implies a deficient where-process for the computation of target coordinates, which affects right-sided saccades only when a vector-inversion is required.

We recently identified an additional deficit in neglect that does not depend on the direction of the saccade. In many daily situations saccades must quickly be re-programmed in response to changing environmental conditions. When two stimuli are presented in fast sequence at different positions or when participants make a corrective saccade following a primary error saccade the latency of the second saccade is much shorter than the latency of the first saccade (Becker and Jürgens, 1979; McPeek et al., 2000). This finding indicates that the second saccade is planned during the execution of the first saccade or even before the first saccade begins. We examined in six neglect patients the latencies of primary saccades that had been directed erroneously to a distracter presented in the left or right hemifield, and of corrective saccades that subsequently re-directed gaze to the target (Ptak et al., 2010). Regarding the proportion of corrective saccades directed to the target in the opposite hemifield to the distracter, we observed the expected hemifield asymmetry in neglect patients. That is, patients made more corrective saccades to targets in the left hemifield than to targets in the right hemifield. However, when considering the latency of corrective saccades we found that while these were significantly shorter than the primary saccade in control participants, no such shortening of latency was observed in neglect patients, whether the saccade sequence involved a left-right or a right-left movement. This finding suggests a bilateral impairment of concurrent saccade programming in patients with left neglect. We related this finding to the impairment of neglect patients to detect the second of two stimuli presented in rapid succession (attentional blink), which has previously been observed even for stimuli presented at fixation (Husain et al., 1997). Concurrent saccade programming requires a selection of the correct stimulus by attention while the primary saccade is prepared. A prolonged attentional blink may thus delay visual analysis following detection of the primary saccade target and the second saccade will therefore be delayed until the secondary target has been selected by attention. Thus, the spatially non-lateralized increase of the attentional blink may explain the bilateral impairment of concurrent saccade programming.

## Eye movements in scene perception

Oculomotor studies of scene perception generally measure sequences of saccades while participants explore graphical patterns or photographs of natural scenes. One early study examined how hemianopic and neglect patients explored simple patterns and found that the former explored the impaired (contralesional) side longer, while the latter directed their gaze more often to the ipsilesional side (Ishiai et al., 1987). The failure of neglect patients to explore the contralesional side does not depend on visual stimulation, as it is also observed when they explore their environment in the dark (Hornak, 1992; Karnath and Fetter, 1995).

**Figure 3:**
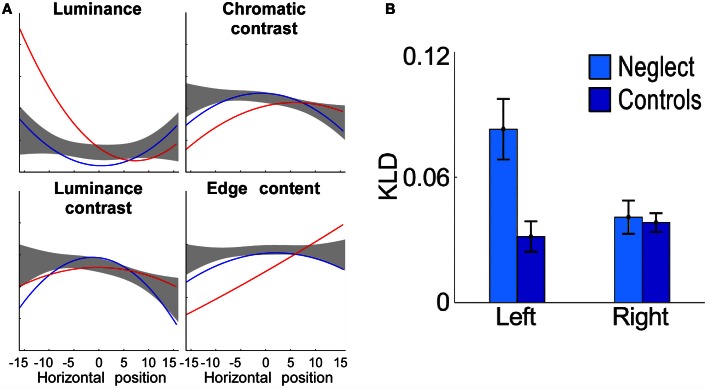
**(A)** Local image content as a function of the horizontal position of fixations, computed for patients with neglect (red line) and control patients (blue line). Note the strong deviation of luminance and edge content in the data of neglect patients as compared to control data (grey area: 99% confidence interval of healthy controls; adapted from Ptak et al., 2009). **(B)** Fixated image regions located on the left have greater low-level feature content than regions on the right for neglect patients but not for healthy controls (KLD refers to Kullback-Leibler Divergence, a measure of the difference between the distributions of low-level features in the image and fixated low-level features; adapted from Ossandón et al., 2012). With permission from Elsevier.

Concerning the form of the horizontal distribution of fixations, Behrmann et al. (1997) reported in neglect patients a gradual decrease of fixation density from the right-most to the left-most location, supporting an orientational gradient account of neglect (Kinsbourne, 1993). However, this finding is an exception and appears to be due to the small display used in this study. Several other studies showed that the distribution is bell-shaped, though the median is significantly shifted ipsilesionally (Karnath et al., 1998; Müri et al., 2009; Ptak et al., 2009; Machner et al., 2012). Thus, the centre of the distribution (measured in terms of the point of maximal density of fixations) is located right of the centre of the body, but further to the right the fixation density decreases. An additional finding is that the amplitude of saccades during ocular exploration is smaller in neglect patients than healthy controls or control patients without neglect—irrespective of saccade direction (Ptak et al., 2009). This finding contrasts with the amplitude of saccades to isolated visual targets in simple saccade paradigms, which is comparable to controls for ipsilesional targets and hypometric for contralesional targets (see section Eye Movements in Basic Saccade Paradigms). One possibility to explain this discrepancy is that patients produce hypometric leftward saccades and normal rightward saccades in conditions that favor stimulus-driven processing, whereas when the task requires voluntary processing saccades are hypometric in all directions (Niemeier and Karnath, 2003). According to Niemeier and Karnath (2003) the reason for this pattern is that only during voluntary exploration are eye movements guided by the fundamental spatial bias underlying neglect. Alternatively, during free visual exploration the eye movements of neglect patients might be captured by irrelevant nearby perceptual details. This interpretation suggests that the local image characteristics guiding exploration are qualitatively different for neglect than control participants. We examined this prediction by measuring the local content of small image patches drawn around each fixation that was made by healthy participants, brain-injured patients without neglect and neglect patients while they freely explored images depicting natural scenes (Ptak et al., 2009). Previous studies have shown that healthy subjects preferentially look at image regions characterized by high local contrast (Reinagel and Zador, 1999; Parkhurst and Niebur, 2003) and edge content (Tatler et al., 2005). We therefore computed for each image patch the local intensity, chromatic contrast, luminance contrast and edge content and compared these to randomly chosen patches. The relation between feature content and the horizontal position of a fixated patch was mostly nonlinear, indicating that the content of image regions sampled by gaze either increased or decreased toward the edges of the display. Interestingly, neglect patients showed significant deviations from both control groups for luminance and edge content (Figure 3). Though the selection of image regions in the right hemifield was based on the same local features as in healthy participants, neglect patients looked preferentially to regions of high local luminance in the left hemifield. In parallel, the edge content of fixated areas gradually increased from left to right, showing that the gaze of neglect patients was biased toward local edge information in the right hemifield. Similarly, Ossandón et al. (2012) found that neglect patients directed their gaze to contralesional locations with higher feature content than healthy controls, whereby feature content was a compound measure of local luminance contrast, color contrast and edge content (Figure 3). In addition, a recent study using videos of real-world scenes revealed that saccades of neglect patients directed contralesionally landed on regions of increased dynamic contrast, a measure reflecting the intensity of local motion (Machner et al., 2012). Together, these findings show that only regions with increased local saliency (in terms of higher luminance or local motion) attract the gaze of neglect patients to the left of a visual scene. In addition, these patients are biased toward looking at regions of particularly high edge content in the right part of the scene, which is reminiscent of their tendency to orient attention toward local features (Delis et al., 1986).

## Impairments of saccade programming following bilateral parietal damage

Bilateral damage to the posterior parietal cortex leads to a severe constriction of attention to a single item (simultanagnosia), impaired control of intentional eye movements (oculomotor apraxia) and spatial errors in visually-guided reaching and pointing (optic ataxia), a combination of symptoms first described by Bálint (1909). Compared to investigations of patients with unilateral damage, studies measuring eye movements of patients with bilateral parietal damage are rare. One reason is the low probability of bilateral damage affecting both parietal lobes. The other reason is that the calibration of eye trackers requires the stable fixation of visual targets presented sequentially at different positions, and thus depends on the subjective judgment of the patient. However, the severe eye movement disorder that results from bilateral posterior parietal damage prevents the detection and stable fixation of the calibration stimuli. In many reports the characterization of oculomotor apraxia therefore relied on clinical observations. The primary characteristics of oculomotor apraxia are an inability to shift gaze to objects in the visual periphery, sometimes resulting in apparently random eye movements, and an impairment of fixation with the failure to maintain fixation on a stimulus, or conversely the failure to move gaze from the current focus of fixation (Bálint, 1909; Holmes and Horrax, 1919; Damasio, 1985; Rafal, 1997; Rizzo and Vecera, 2002).

We are only aware of one study that analysed quantitatively saccadic programming of a patient with simultanagnosia following bilateral inferior parietal damage using basic saccade paradigms. Nyffeler et al. (2005) measured at three different time points following onset of the disease saccade latencies in the gap and overlap paradigm of a patient presenting the severe constriction of attention characteristic of simultanagnosia. Interestingly, in the gap task the patient had slightly *shorter* (though not significantly) saccade latencies than healthy controls, while in the overlap task her latencies were up to the double of the latency of controls (508 ms versus 260 ms). Increased saccade latencies were observed at all three testing sessions, though in the same interval visual exploration of simple line drawings had recovered to a level similar to healthy participants.

**Figure 4:**
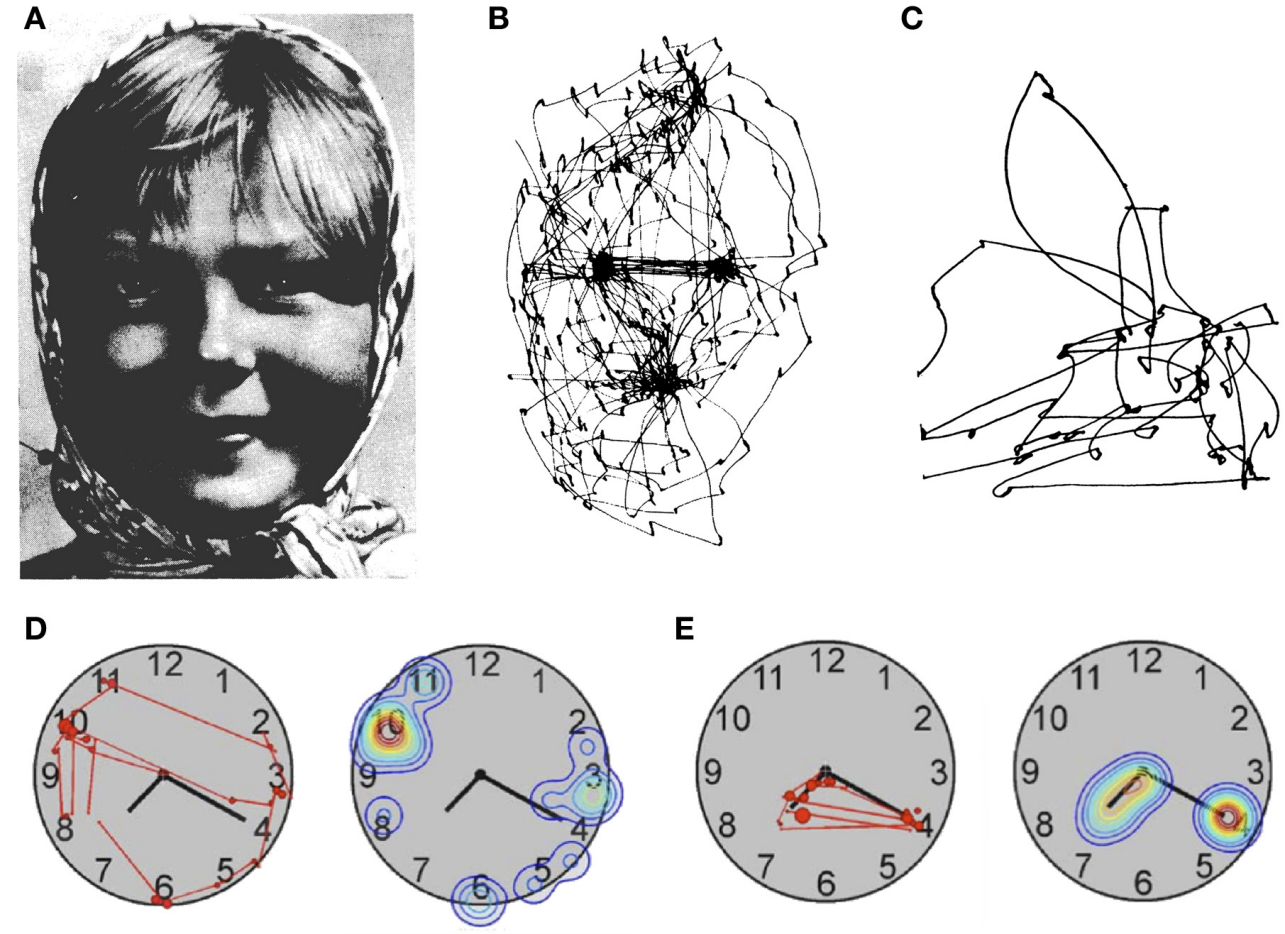
**Patterns of visual exploration in simultanagnosia. (A)** Portrait used by Yarbus (1967) when studying face exploration. **(B)** Scan-path of a healthy subject and **(C)** of a patient with simultanagnosia when exploring the picture shown in **(A)**. **(D)** Scan-path and fixation-density plot from a patient with simultanagnosia and **(E)** a healthy subject exploring a clock-face [**A** and **B**: adapted from Yarbus (1967); **C**: adapted from Luria et al. (1963); **D** and **E**: adapted from Nyffeler et al. (2005)] with permission from Elsevier.

A much earlier eye tracking study using a method developed by Yarbus (1967) had already investigated visual scanning in a patient with simultanagnosia (Luria et al., 1963). The authors noted that the patient had normal fixation of an isolated light in the dark as well as normal smooth pursuit eye movements of a single target in regular motion. In contrast, eye movements were essentially random when the task required visual scanning, such as when the patient was asked to perform simple saccades between two stimuli or when observing the image of a face (Figure 4). Another patient examined by Girotti et al. (1982) also exhibited spatially disorganized eye movements and was only able to fixate a visual stimulus after repeated erratic eye movements. In contrast to Luria's patient however, their patient was also unable to follow visually a moving target and could not voluntarily generate saccades on verbal command, suggesting a generalized impairment of eye movement programming. The patient of Nyffeler et al. (2005) showed abnormal scanning of horizontally aligned line drawings and of the picture of a clock face (Figure 4). When asked to read the clock she inspected uninformative numbers irrespective of the position of the hands instead of fixating the clock hands and the corresponding numbers as control participants did. Two other simultanagnosic patients (Dalrymple et al., 2011, 2013) failed to fixate the informative eye regions in social scenes, while making many fixations on irrelevant stimuli.

According to Bálint oculomotor apraxia (which he referred to as “psychic paralysis of gaze”) in his patient was secondary to the pathological constriction of attention to a single object, and several authors agree with this interpretation (Damasio, 1985; Rafal, 1997; Rizzo and Vecera, 2002). However, the precise relation between oculomotor apraxia and the impairment of visual attention is unclear. For example, some patients may show erratic and disorganized fixation patterns yet are able to extract a significant part of the information contained in complex visual displays (Jackson et al., 2006). Clavagnier et al. (2006) did not observe qualitative differences in the scanning pattern of simultanagnosia patients for stimuli they could identify compared to items they failed to recognize. Similarly, Dalrymple et al. (2009) noted that eye movements did not predict performance of their simultanagnosic patient in identification of hierarchical stimuli. Thus, contrary to Bálint's hypothesis these observations suggest a rather loose relation between simultanagnosia and oculomotor apraxia.

A possible solution to this inconsistency is suggested by an observation made by Rizzo and Hurtig (1987). These authors examined three patients with simultanagnosia and found a dissociation between eye movements and subjective awareness of visual objects. For example, the patients reported disappearance of a light stimulus while they were directly fixating it, suggesting a spontaneous drift of visual awareness from the object at the centre of gaze. Studies with healthy participants show that the relation between overt attention (gaze position) and covert attention (attentional focus) is best described by an interdependence hypothesis, which postulates that attention might be shifted without shifting the eyes, but that eye movements and attention cannot fully dissociate (e.g., the eyes cannot move in one direction while at the same time attention moves in the other direction; Remington, 1980; Hoffman and Subramaniam, 1995). However, the subjective disappearance of a fixated object from awareness in simultanagnosia may reflect pathological fading of visual representations following prolonged viewing, akin to the Troxler effect in healthy observers (Rizzo and Hurtig, 1987; Farah, 1990). The question arising from these observations is: what could explain the observed dissociations between object identification in complex displays and the erratic scanning pattern of patients with simultanagnosia? In our view the answer depends on a better specification of the variables that determine the seemingly chaotic pattern of visual exploration. The clock-reading data of Nyffeler et al. (2005; Figure 4) suggest that irrelevant perceptual details capture the gaze of patients with simultanagnosia. Based on this observation the authors proposed that during visual exploration the gaze of these patients is guided by bottom-up visual saliency rather than the information content of specific regions of the image. Such impairment would result in many erroneous saccades to irrelevant stimuli, and together with attempts of the patient to produce compensatory eye movements for her/his drifting awareness, would lead to the seemingly chaotic scanning pattern observed in several studies. A complementary observation was made by Dalrymple et al. (2013), whose simultanagnosic patient switched from a seemingly chaotic to an ordered fixation pattern (fixating face and eye regions) when asked to infer attentional states of people in social scenes. This observation shows that simultanagnosic patients may be able to use top-down strategies to guide eye movements but do not use them spontaneously (see also Jackson et al., 2009). Together, these findings suggest that deficits of visual scanning in simultanagnosia reflect at least two factors: increased bottom-up capture by salient, but irrelevant information, together with underuse of preserved top-down strategies. This combination of deficits is in line with recent proposals that the guidance of eye movements in natural vision not only depends on bottom-up saliency factors, but also on feature relevance and associated reward values (Tatler et al., 2011).

## Understanding impairments of saccade programming following parietal damage

Traditionally, eye movement disorders following parietal damage have been interpreted in terms of an underlying impairment of visual attention (Bálint, 1909; Holmes and Horrax, 1919; Damasio, 1985; Rafal, 1997). This interpretation is based on the observation that oculomotor apraxia always occurs together with visual attention deficits and the fact that patients are able to execute eye movements on command and to non-visual targets (note however, that these observations are mainly anecdotal). The strong association between visual attention and saccade programming is also observed in psychophysical studies with healthy participants. For example, subjects are better at discriminating a visual target at the location of an upcoming saccade than at alternative locations (Deubel and Schneider, 1996), and they find it impossible to program a saccade to one stimulus while simultaneously making a perceptual decision of another stimulus (Kowler et al., 1995). In agreement with this strong functional coupling between attention and saccades, neuroimaging studies show a close overlap of activations at the dorsal fronto-parietal convexity that are related to covert shifts of attention and activations related to saccades (see Grosbras et al., 2005 for a meta-analysis of functional imaging studies). Current accounts of PPC function (in particular, of those areas involved in visual attention and saccade programming, such as area LIP) propose that this region integrates bottom-up saliency information with top-down task-related signals into an attentional priority map of the environment (Gottlieb, 2007; Bisley and Goldberg, 2010; Ptak and Fellrath, 2013). The priority map is believed to act according to an “all-or-none” rule, in that the stimulus with the highest current saliency will automatically be selected by attention (Koch and Ullman, 1985). Indeed, intraparietal damage leads to a deficit of target selection when stimuli compete for attentional resources (Gillebert et al., 2011), and disconnection of the PPC from the premotor cortex (including the FEF) may disrupt top-down signals biasing attentional selection in spatial neglect (Bays et al., 2010; Ptak and Schnider, 2010).

The visual scanning behavior of patients with unilateral or bilateral parietal damage is in agreement with the notion of attentional priority. For example, the rightward bias of the first saccade during scene exploration in neglect patients and their tendency to look to the more ipsilesional of two simultaneously presented stimuli are compatible with a bias in the priority map favoring right-sided locations. In addition, the finding that patients with neglect and simultanagnosia preferentially fixate visually salient rather than particularly informative regions suggests a failure to integrate bottom-up and top-down attention signals.

However, the saccade studies discussed above also present some findings that are difficult to reconcile with an attentional account. For example, the relation between erratic scanning behavior of patients with simultanagnosia and their capacity to extract information from the image is not straightforward, and dissociations between both measures may be observed (Clavagnier et al., 2006). Even more puzzling are observations of bilateral deficits in saccade performance following unilateral parietal damage leading to a spatially lateralized deficit of visual attention. We propose that at least some of these deficits reflect subcortical interactions in the oculomotor system that are only revealed when top-down modulation is altered due to cortical damage. One such observation is the pathological bias in oculomotor responses favoring stimuli presented at fixation that characterizes left neglect (Ptak et al., 2007). Trying to explain this bilateral bias in terms of attentional failures is problematic. Some authors have interpreted the overlap effect in terms of impaired disengagement of attention from fixation (Fischer and Breitmeyer, 1987), and a multi-modal disengagement deficit is one of the core attentional components of spatial neglect (Posner et al., 1987; Losier and Klein, 2001; Golay et al., 2005). However, neglect patients only fail to disengage attention from ipsilesional stimuli, and the deficit generally appears in manual reactions. In contrast, the increased overlap effect was clearly not lateralized and was restricted to oculomotor responses. This finding is coherent with a model of spatial neglect that posits mutual inhibitory interactions between the parieto-occipital cortex and the superior colliculus (Sprague, 1966; Rafal, 2006). In animals, impaired orienting responses toward stimuli contralateral to a parieto-occipital lesion are restored if the contralateral colliculus is functionally inactivated (a finding known as the “Sprague-effect”; Sprague, 1966; Payne et al., 1996). The Sprague-effect suggests that parieto-occipital cortex normally exerts a facilitatory influence on the ipsilateral and an inhibitory influence on the contralateral colliculus. An important feature of the superior colliculus is its subdivision in two functionally distinct regions: the rostral colliculus contains neurons that discharge when a stimulus in the central ~2° of the visual field is actively fixated (Munoz and Wurtz, 1993), while neurons in the caudal colliculus show activity related to the preparation and execution of saccades (Dorris et al., 1997). Stimulation of fixation neurons in one colliculus activates fixation neurons and deactivates saccade-related neurons in the other colliculus (Munoz and Istvan, 1998), suggesting that both colliculi work as a unit during active fixation. Thus, following a right cortical lesion, a functionally disinhibited left colliculus would stimulate the fixation zone in the right colliculus, which would lead to increased fixation activity. The bilateral increase of saccade latency following a foveal distracter can thus be understood in terms of functional interactions between the right parietal lobe and the two colliculi.

This tentative explanation remains hypothetical as long as no direct evidence exists that the Sprague-effect applies to spatial neglect in humans (though positive results have been presented by Weddell, 2004). Nevertheless, interactions between fixation and saccadic activity may be pathologically increased following cortical damage, and they therefore provide a logical alternative to attention deficits for the explanation of some “low-level” oculomotor impairments (this might also apply to the remote distracter effect or the global effect). In sum, eye movement deficits following focal parietal damage may reflect the importance of this region for saccade planning under conditions of sensory competition as well as functional impairments of remote structures of the oculomotor network lacking top-down modulation.

### Conflict of interest statement

The authors declare that the research was conducted in the absence of any commercial or financial relationships that could be construed as a potential conflict of interest.
